# Resting-State Brain Organization Revealed by Functional Covariance Networks

**DOI:** 10.1371/journal.pone.0028817

**Published:** 2011-12-13

**Authors:** Zhiqiang Zhang, Wei Liao, Xi-Nian Zuo, Zhengge Wang, Cuiping Yuan, Qing Jiao, Huafu Chen, Bharat B. Biswal, Guangming Lu, Yijun Liu

**Affiliations:** 1 Department of Medical Imaging, Jinling Hospital, Nanjing University School of Medicine, Nanjing, Jiangsu Province, China; 2 Key Laboratory for NeuroInformation of Ministry of Education, School of Life Science and Technology, University of Electronic Science and Technology of China, Chengdu, Sichuan Province, China; 3 Laboratory for Functional Connectome and Development, Institute of Psychology, Chinese Academy of Sciences. Beijing, China; 4 Phyllis Green and Randolph Cowen Institute for Pediatric Neuroscience, New York University Child Study Center, New York, New York, United States of America; 5 Department of Radiology, UMDNJ New Jersey Medical School, Newark, New Jersey, United States of America; 6 Department of Psychiatry, University of Florida, Gainesville, Florida, United States of America; Tokyo Metropolitan Institute of Medical Science, Japan

## Abstract

**Background:**

Brain network studies using techniques of intrinsic connectivity network based on fMRI time series (TS-ICN) and structural covariance network (SCN) have mapped out functional and structural organization of human brain at respective time scales. However, there lacks a meso-time-scale network to bridge the ICN and SCN and get insights of brain functional organization.

**Methodology and Principal Findings:**

We proposed a functional covariance network (FCN) method by measuring the covariance of amplitude of low-frequency fluctuations (ALFF) in BOLD signals across subjects, and compared the patterns of ALFF-FCNs with the TS-ICNs and SCNs by mapping the brain networks of default network, task-positive network and sensory networks. We demonstrated large overlap among FCNs, ICNs and SCNs and modular nature in FCNs and ICNs by using conjunctional analysis. Most interestingly, FCN analysis showed a network dichotomy consisting of anti-correlated high-level cognitive system and low-level perceptive system, which is a novel finding different from the ICN dichotomy consisting of the default-mode network and the task-positive network.

**Conclusion:**

The current study proposed an ALFF-FCN approach to measure the interregional correlation of brain activity responding to short periods of state, and revealed novel organization patterns of resting-state brain activity from an intermediate time scale.

## Introduction

Interest in investigating spontaneous low-frequency fluctuations in resting-state brain activity is steadily growing (see [1], [2] for systematic reviews). It is postulated that this intrinsic activity reflects the brain's “dark energy” consumption at rest [2], [3], and is closely relevant to the perceptive or cognitive processes by sharing similar functional topography with specific task-induced brain activity. The intrinsic activity is known to consists of various large-scale intrinsic connectivity networks (ICNs) [4], [5], observed by resting-state functional magnetic resonance imaging (rs-fMRI) [1], [6], [7]. By measuring low-frequency (<0.1 Hz) fluctuations in blood oxygenation level dependent (BOLD) signal, rs-fMRI has proven to be a powerful tool for exploring brain function and its clinical implications [2], [8]. The most widely used technique for depicting ICNs is to calculate the temporal correlation of the BOLD time series between two brain regions (i.e., TS-ICN). Moreover, another common technique, independent component analysis, has mapped the resting-state networks through a data-driven analysis manner, repeats the topographic properties of these ICNs [9], [10]. Studies of TS-ICN have unraveled the organization patterns of resting-state brain activity [2], [11]. On a local scale, the resting-state brain can be hierarchically partitioned into several network modules [2], [9], [12], [13]. From view of global scale, the brain consists of two competitive brain network systems: the default-mode network (DMN) and the task-positive network (TPN) [5], [14]. Such a functional dichotomy has been demonstrated high reliability across participants and imaging centers in the 1000 Functional Connectomes Project [15]. Moreover, the subsequent studies have also investigated the relationships among the DMN, TPN and the primary sensory networks [16], [17]. It has been reached a consensus that the DMN may coordinate activity in the other brain networks [3].

Following the TS-ICN approach, a few studies have also demonstrated inter-regional relationship characterized by structural covariances across subjects. Mechelli et al. [18] observed that brain regions covary in gray matter volume across subjects, suggesting a structural covariance network (SCN). The association between SCNs and age was reported in subsequent studies [19], [20], [21]. Moreover, using cortical thickness and graph theoretic analysis, He et al. [22] revealed the large-scale whole brain SCN exhibiting the ‘small-world’ phenomena. SCNs have been interpreted as inter-regionally coordinated structural variances reflecting long-duration effects of brain development or plasticity [18], [19]. Interestingly, noting the similarity of spatial pattern and developmental effect between ICNs and SCNs, recent studies have suggested that SCNs reflect shared long-term trophic influences within functionally synchronous systems (i.e., the ICNs) [19]. However, caution is needed when concluding such relationships between ICNs and SCNs, since these are not characterized by the same-level covariance. Specifically, ICN is calculated as the temporal or across-time covariance in function (i.e., BOLD signal) between regions, whereas SCN reflects across-subject covariance in cross-sectional structure (i.e., morphological data). Currently, there exists no physiological mechanism connecting ICN and SCN analyses. Accordingly, in this work we describe networks revealed by the across-subject covariance in function (i.e., functional covariance network, FCN) based on BOLD-fMRI data, and propose these FCNs as a means to bridge the gap between ICN and SCN.

Individual measures of the amplitude of low frequency fluctuations (ALFF) in resting-state brain activity can serve as a functional measure to compute FCNs. ALFF has been proven to be a reliable index of local intrinsic brain activity [6], [23], [24], [25], which is defined as the total power within a low-frequency range (e.g., 0.01–0.1 Hz) of rs-fMRI signals. ALFF and PET measures, which quantifies resting brain's metabolism during a short period of time [26], exhibit highly consistent spatial patterns [23], [24], [25]. ALFF is capable of interrogating both normal [24], [27] and abnormal brain function [23], [28], [29]. Most recently, individual variability (i.e., across-subject covariance) in ALFF has been linked to those in neural activation and behavior [30], suggesting it may be well suited for FCN analysis.

In the present study, we examined FCNs by calculating inter-regional correlation of ALFF across 310 subjects (i.e., ALFF-FCN) to map the brain network organization patterns corresponding to the DMN, the TPN and the sensory network [5], [16]. We chose three sets of ‘seed’ region of interests (ROIs) for ALFF-FCN analyses: (1) DMN seeds including posterior cingulate cortex (PCC), dorsal medial prefrontal cortex (DMPFC) and left angular gyrus (lAG) [4], [5], [14]; (2) TPN seeds including right dorsal lateral prefrontal cortex (rDLPFC), left intraparietal lobular (lIPL) and left frontal eye field (lFEF) [4], [5]; (3) sensory network seeds including left primary sensory cortex (lSC), left primary visual cortex (lVC) and left auditory cortex (lAC) [16], [17]. We aim to: 1) reshape the brain network organization patterns using ALFF-FCN means building on the findings of ICNs; 2) examine the consistency and variability of spatial patterns across voxel-based morphometry SCN (VBM-SCN), TS-ICN and ALFF-FCN; 3) gain more understanding of the nature of human brain network organization from views of local and global scales.

## Results

### Parallel observations of the FCNs, ICNs and SCNs

**Figure 1:**
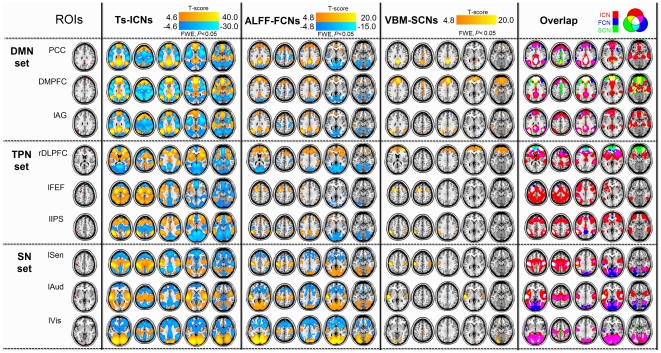
Brain network pattern mapped by ALFF-FCN, TS-ICN and VBM-SCN techniques. All ALFF-FCNs, TS-ICNs and VBM-SCNs showed significantly positive correlation (warm color). Networks from each ROI presented autocorrelation and contralateral homotopic regions, and largely overlapped across the three network approaches. The ALFF-FCNs and TS-ICNs had wider lateral distributions than VBM-SCNs. Both the ALFF-FCN and TS-ICN measures, but not the VBM-SCN measure, yielded significantly negative correlation networks (cold color).

All ALFF-FCNs, TS-ICNs and VBM-SCNs showed widespread network pattern with significantly positive correlation. The results of TS-ICNs and VBM-SCNs were both consistent with the previous studies [5], [16], [19]. As shown in Figure 1, networks from each ROI presented autocorrelation and contralateral homotopic regions, and largely overlapped across the three network approaches. The ALFF-FCNs and TS-ICNs had wider lateral distributions than VBM-SCNs. Both ALFF-FCN and TS-ICN, but not the VBM-SCN, yielded significantly negative correlated (i.e., anti-correlated) networks.

### Hierarchical organization of brain networks

**Figure 2:**
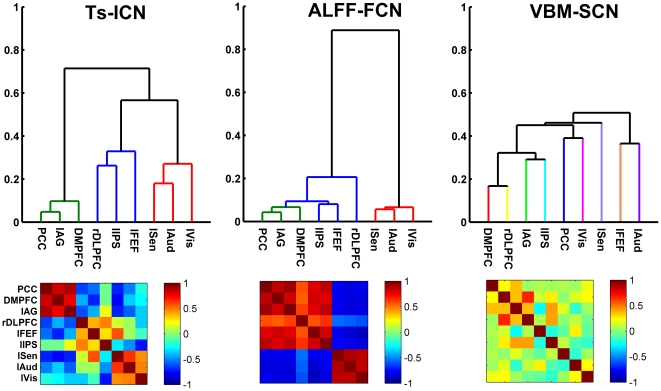
Hierarchical clustering analysis of ALFF-FCNs, TS-ICNs and VBM-SCNs. Hierarchical clustering analysis was based on the spatial correlation maps of nine ROI-based network maps for three network analyses approaches. As shown in root of the dendrogram of ALFF-FCNs, there are two partition systems (DMN combining TPN and SN). While at the root of the dendrogram of the ICN-based network, we observed three partition systems including the DMN, TPN and SN, separately. There are no obvious partition systems observed in the SCN-based network.

**Figure 2** shows the results of the hierarchical clustering of the spatial correlation maps of nine ROI-based network maps for three network analyses approaches. At an arbitrary threshold of clustering distance = 0.4, we can find that in the root of the dendrogram of ALFF-FCNs, there are two partitions (DMN integrating with TPN, and SN). While in the root of the dendrogram of the ICN-based network, we observed three partition systems including the DMN, TPN and SN, separately. However, there are no obvious partition systems observed in the SCN-based network.

### Modular and system patterns of brain networks

**Figure 3:**
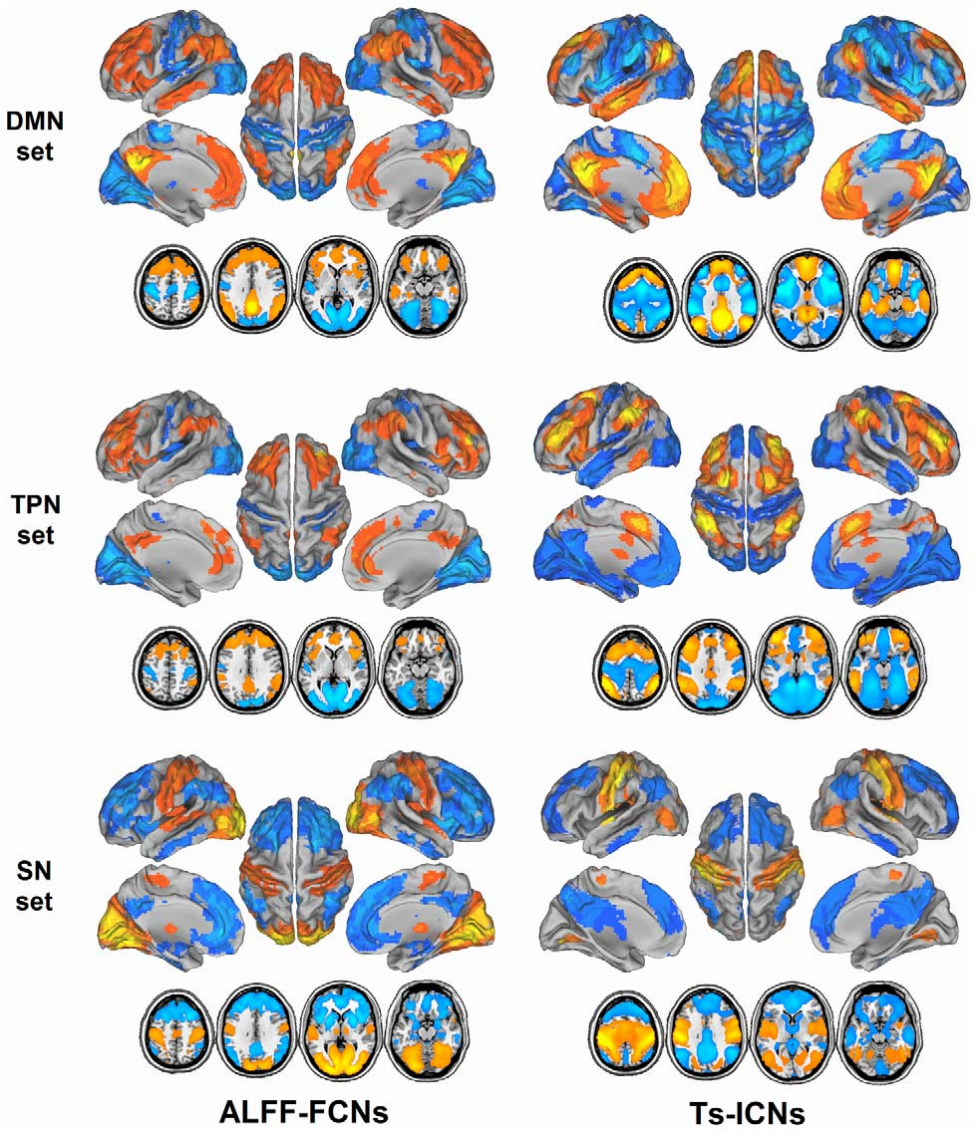
Network patterns of ALFF-FCNs and TS-ICNs at modular level. Intra-ROI set conjunction analyses revealed modularity in TS-ICNs and ALFF-FCNs.

Clustering analysis and intra-ROI set conjunction analyses revealed the network modularity in the TS-ICNs and ALFF-FCNs. Firstly, the positive network in one ROI set showed similar spatial pattern (Figure 2), conjunction analyses showed widely spatial overlap in each network ROI set (Figure 3). In addition, a notable finding was the different patterns of the DMN and TPN modules between TS-ICNs and ALFF-FCNs. In line with the previous studies, in TS-ICNs, the DMN module was opposite to the TPN module with anti-correlations [5], [16]. ALFF-FCNs include the two main modules including DMN/TPN module and anti-correlated SN module. Hence, at the cutoff of 0.4, clustering analysis revealed three network systems in TS-ICNs, but two network systems in ALFF-FCNs.

**Figure 4:**
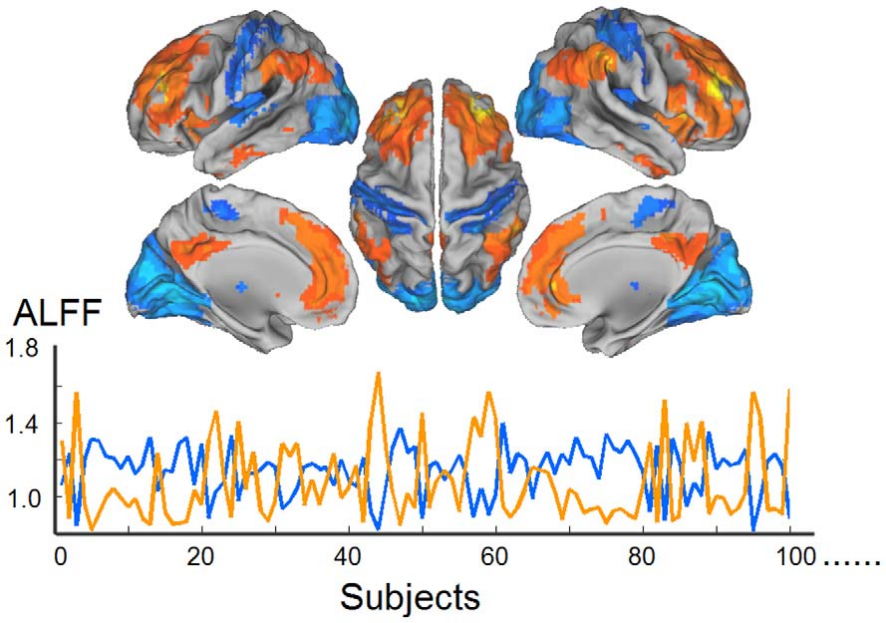
Anti-correlation of ALFF values across subjects between the systems in ALFF-FCN dichotomy. Inter-ROI set analysis of ALFF-FCNs revealed a network dichotomy, which consisting of a high-level cognitive network system covering the DMN and TPN regions, and a low-level cognitive network system covering sensory regions The ALFF values within the high-level cognitive system (warm color) negatively correlated with those of the low-level cognitive (perceptive) system (cold color) (*r* = −0.822, *P* = 3.697×10^−17^).

The inter-ROI set conjunction analyses of ALFF-FCNs further revealed a network dichotomy with anti-correlation (**Figure 4** and **Table 1**). The regions showing negative correlation in the DMN and TPN modules were those showing positive correlation in the SN module. Hence, the brain could be divided into a high-level cognitive network system covering the DMN and TPN regions, and a low-level network system covering sensory regions. Moreover, significantly negative correlation (*r* = −0.822, *P* = 3.697×10^−17^), but no difference (*t* = 0.605, *P* = 0.545) was found between the mean ALFF values within the two network dichotomies (high-level cognitive areas: 1.109±0.125; the lower-order cognitive system: 1.114±0.194) (**Figure 4**).

**Table 1:** Network dichotomy revealed by ALFF-FCN.

Brain areas	x, y, z (MNI)	T-value	Voxels
Higher order cognitive system			
PCC/Pcu	9, −37, 40	6.06	435
l Angular gyrus	−57, −34, 40	7.80	539
l IPS	−60, −31, 37	7.63	
r Angular gyrus	57, −61, 28	6.60	800
r IPS	60, −34, 43	9.56	
VMPFC	6, 44, −11	6.84	6145
DMPFC	−6, 38, 31	7.31	
l DLPFC	−45, 35, 1	8.71	
r DLPFC	48, 35, 4	7.93	
Lower order cognitive system			
l Auditory cortex	−60, −15, 3	5.02	104
r Auditory cortex	57, −15, −3	3.26	28
l Visual cortex	−21, −87, −9	5.35	3055
l MT+	−42, −72, 6	4.69	
r Visual cortex	15, −81, −3	4.94	
r MT+	45, −78, −6	4.28	
l Sensory cortex	−51, −24, 48	6.20	542
r Sensory cortex	51, −18, 42	3.90	337
l Thalamus	−9, −21, 9	3.15	48
r Thalamus	12, −27, 12	2.94	70

Abbreviations: PCC: post cingulate cortex; Pcu: Precuneus; l IPS: left intraparietal lobular; l: left; r: right; VMPFC: ventral medial prefrontal cortex; DMPFC: dorsal medial prefrontal cortex; DLPFC: dorsal lateral prefrontal cortex; MT+: middle temporal plus; FEF: frontal eye field.

## Discussion

Based on a large sample of resting-state fMRI datasets, the present study mapped neuro-anatomic patterns of functional covariance networks of different brain systems by correlation analysis of ALFF across subjects (ALFF-FCNs), which supplied a novel way to analyze resting-state fMRI data. By comparing with the TS-ICN and VBM-SCN approaches, the ALFF-FCNs showed specific pattern of network organization in human brain, indicating they are physiological and metabolic in nature. The results extend our insight into the human brain network organization.

### FCNs as the bridge between ICNs and SCNs

Current brain network approaches mostly rely on two correlation techniques [35], [36]. The first is intervoxel time-series correlation in individual data, such as applied in the functional connectivity measurements of EEG/MEG and the majority of fMRI studies, as well the time-series ICN measurement in the current study. The second means is interregional correlation of a given imaging index across subjects, such as the network mapping in PET [37], [38], [39], [40], SPECT [41], SCNs [18], [19], [22], [42] and ALFF-FCNs measurements. Commonly, these cross-sectional data were required to be collected from a large-amount of homogenous subjects [39] (i.e., perhaps in an ideal limit, consisting of multiple sessions from a single subject).

Time-scale difference might be the foremost assumption for understanding the network properties between these two correlational approaches, although the evidences are far from enough in the current work. The time-series correlation measures the instantaneous variability of brain activities, while the correlation across subjects may be driven by long-duration effects of brain activities [18], [19]. TS-ICN measures coherent BOLD variations (low-frequency BOLD fluctuations: 0.01∼0.08 Hz) over very short time scales (corresponding periods: 100 s∼12.5 s) through a high sampling rate (0.5∼3 s for typical repetition time of fMRI data acquisitions), resulting from instantaneously neural activities in individual [1], [8]. While SCN measures the variances of cortical structures concerning brain development or plastic, since gray matter structures are framed by longer-duration (years) effects of gene and circumstance [18], [19], which might reflect the brain organization at largest time scales (years). Because ALFF measures the total level of neural activity during a short period of time (5∼10 mins scans), and can be considered to be comparable with resting PET [23]. Hence the ALFF-FCN provides a tool to show the brain organization at interim time scale window of particular brain state. As analogue to the network analysis in PET or SPECT studies, the positive and negative correlations in ALFF-FCN may indicate the coherence and inhibition of interregional brain activity [41]. However, the ALFF-FCN study revealed more information about the brain network organization property than the PET of network analysis [40].

The present study revealed hierarchical organization properties of human brain from individual network, module and system levels through the three network approaches. Firstly, at individual network level, networks produced with identical seed region across TS-ICN, ALFF-FCN and VBM-SCN showed similar spatial pattern and largely overlapped. Regarding the previous finding that the SCNs were shown to recapitulate the canonical ICN topologies [19], [42], the results indicate that the individual networks in the human brain are robustly organized with consistent spatial patterns, regardless of the analysis procedure used.

Although substantial overlaps were observed between the TS-ICN, ALFF-FCN, and VBM-SCN, there were differences between the covariance based maps. For example, the TS-ICN, in which the statistic analyses were performed at the group level, had the greatest spatial distribution. The VBM-SCN, although has the same statistical level with ALFF-FCN, showed the most limited spatial distribution. The difference of spatial patterns of these three methods may attribute to the difference of data modalities (i.e., functional and structural), covariance level or time scales.

When seeding at the three ROIs in one ROI set, the ALFF-FCNs showed consistent spatial pattern with large overlap among themselves. The intra-ROI set conjunction analysis revealed network modules of the DMN, TPN and SN. Meanwhile, the spatial pattern of each network module mapped by ALFF-FCN was similar to that revealed by TS-ICN. Various studies based on TS-ICNs have found different patterns of brain network modules. Commonly, the DMN module is robustly detected, but the others have different networks classified manners [2], [9], [12]. Although a few studies have revealed that the networks in the SN system, i.e., the somatosensory network, auditory network and visual network present separate modules [2], [9], these networks are mostly detected with an integrative pattern under task- or resting state [12], [16], [43]. The present study showed modular nature of SN by spatial conjunction of TS-ICNs and ALFF-FCNs. However, this modular nature was not found in the SCNs.

Interestingly, hierarchical clustering revealed different patterns of networks clustering between the ALFF-FCN and TS-ICN approaches at a system level. ALFF-FCNs and time-series ICNs both demonstrated the feature of network dichotomy with anti-correlations, but there were different dichotomic patterns. For ALFF-FCNs, the dichotomy consists by the high-level cognitive system covering the DMN and TPN regions, and the low-level cognitive system covering all sensory regions. This pattern was consistent with the traditional view of brain partitions [17]. This novel dichotomic pattern was significant different from the one revealed by TS-ICN. Fox et al., have divided the resting state brain network into dichotomy of DMN and TPN [5]. These two TS-ICNs are responsible for the immediately competitive activities of the internal mentation and external goal-oriented task, respectively [5]. Hence, regarding the ALFF-FCN dichotomy which hierarchically consisted of high- and low-level cognitive systems, we tentatively suppose that there is, at least two types of competitive systems organizing the resting-state brain activities. At a small time-scale of instantaneous BOLD time points, the brain activity of the external system (i.e., the DMN) is dynamically opposed to that of the external system (i.e., the TPN) [5]; while at a rather larger time scale of state period, the brain activity of the high-level brain network regions are competitive with that of the low-level network regions. This supposition may be supported by a few of recent findings. At scales of short period of time, the DMN activity can be affected by different lower grade perceptive cognition load, such as the visual [44], auditory [45] and tactile processes [46]. The findings indicate that, the brain network may have different system organization patterns at different time scales.

Another noteworthy finding is the switching of the network roles played by the thalamus. The thalamus often appears in the DMN mapped by time-series ICN [14], [47]. In contrast, in ALFF-FCNs, the thalamus appears excluded from the usual DMN in favor of the sensory network, However, the meanings of this manifestation still need further investigation in future studies.

### Methodological consideration and limitations

Based on the previous findings of the TS-ICNs and VBM-SCNs, this study for the first time investigated the ALFF-FCN by applying the cross-subjects correlation to fMRI data. The ALFF-FCN was considered to represent the profile or pattern of brain organization that underlies a neuro-pathophysiology or particular brain state. The physiological significance of ALFF-FCN is still far from clear. Different cognition-loads during scans were firstly assumed to be one origin of the ALFF variance [27]. However, the limitation of this study is the absence of detailed behavioral data during scan, such as the resting-state questionnaire [48]. Secondly, for excluding the possible bias from the subject variant, a longitude study based on multiple scans of same subjects may be the ideal model for study the covariance in different states. Thirdly, in an intuitive sense, the ALFF-FCN is somewhat similar to the metabolic functional connectivity in PET studies [23]. A recent PET study has investigated the metabolic network architecture at whole brain level by seeding with 70 ROIs, but the results showed that connectivity from each seed region was restricted within a separated network, few network demonstrated wide distributed pattern [40]. Rather small sample of subjects (n = 50) and different imaging modalities may both be the reason for different demonstrations between the metabolic networks and ALFF-FCNs. Future study based on large amount PET data may help clarify the neuro-physiological meaning of ALFF-FCNs. Fourthly, in the paper we adopted global signals regression for seed-based correlation analysis in order to keep in line with the previous studies [5], [19]. It is known that correction for global signals is prone to produce extensive anti-correlations, although there currently remains controversy on the essential of global signals regression [33], [49], [50], [51].

### Conclusion

The present study investigated the functional covariance based ALFF measurement across subjects, and proposed an ALFF-FCN approach to measure the interregional correlation of brain activity responding to short periods of state. By combining ALFF-FCN with TS-ICN and VBM-SCN approaches, we also investigated the brain networks at different time scales, and found the human brain is differently organized at levels of network, module and system. A novel ALFF-FCN dichotomy may suggest that the resting-state brain activity is anti-correlated between the high-level and low-level functional networks across short periods of states at a meso-time scale level.

## Materials and Methods

### Subjects and MRI methods

Our analyses were performed based on two neuroimaging datasets including both structural and rs-fMRI data from total 310 healthy subjects. The first was obtained from the Jinling Hospital at Nanjing University (n = 112). The second was from the State Key Laboratory of Cognitive Neuroscience and Learning at Beijing Normal University (n = 198), which now is part of the “1000 Functional Connectomes” Project (http://www.nitrc.org/projects/fcon_1000/). All participants from the two centers homogenously consisted of 140 males (age: 21.86±1.97 years) and 170 females (age:21.52±1.91 years) young college students. They all had no history of neurological and psychiatric disorders. Written informed consent was obtained from each participant, and the study was approved by the Institutional Review Boards at Jinling Hospital and the State Key Laboratory of Cognitive Neuroscience and Learning at Beijing Normal University. During the resting state, participants were instructed to keep still with their eyes closed but not fall asleep, remain as motionless as possible. MRI data were acquired on same machine type scanners (Siemens 3T Trio) with almost identical protocols. Specifically, functional images (Jinling Hospital/Beijing Normal University) were acquired by using of single-shot gradient echo-echo imaging (GRE-EPI) sequence (repetition time: 2000 ms/2000 ms; echo time: 30 ms/30 ms; slices: 30/33; thickness: 4 mm/3 mm; gap: 0.4 mm/0.6 mm; field of view: 240 mm×240 mm/200 mm×200 mm; in-plane resolution: 64×64/64×64; flip angle: 90°/90°). Structural images (Jinling Hospital/Beijing Normal University) were acquired by using 3-dimensional magnetization-prepared rapid gradient-echo (MPRAGE) sequence (repetition time: 2300 ms/2530 ms; echo time: 2.98 ms/3.39 ms; inversion time: 900 ms/1100 ms; field of view: 256 mm×256 mm/256 mm×256 mm; flip angle: 9°/7°; in-plane resolution: 256×256/256×256) After excluding 10 individual data with artifacts, 300 datasets were employed for subsequent analyses.

### Data preprocessing and analysis

rs-fMRI datasets were processed using a toolkit of DPARSF which synthesizes SPM8 procedures [31]. Functional images were first preprocessed including slice timing correction, realignment of all images to the first image, spatial normalization to Montreal Neurological Institute 152 (MNI152) template with 3×3×3 mm^3^ re-sampling, spatial smoothing with an 8 mm full-width half maximum (FWHM) isotropic Gaussian kernel, and temporal band-pass filtering (0.01–0.08 Hz).

To calculate ALFF measure at each voxel, the time series was transformed to the frequency domain by using fast Fourier transform. The power spectrum was then computed and square root-transformed at each voxel. The averaged square root of activity in the low-frequency band (0.01–0.08 Hz) was taken as the ALFF. The ALFF value of each voxel was standardized by dividing the full-brain mean ALFF values [23], [24], [25].

Structural data were preprocessed using VBM8 implemented in SPM8. The images of each subject were transformed into standard MNI152 space with a 12-parameter affine-only non-linear transformation, and re-sampled to 1.5×1.5×1.5 mm^3^. All images were then segmented into three tissue classes representing gray matter (GM), white matter (WM) and cerebrospinal fluid (CSF). The resultant tissue images were further smoothed with an 8 mm FWHM isotropic Gaussian kernel for subsequent morphometry analyses.

### Network measurements and analyses

To compare architectures of TS-ICNs, VBM-SCNs and ALFF-FCNs, nine spherical cerebral regions with radius of 6.0 mm from previous studies [5], [14], [16], [32] served as seed ROIs. These ROIs were selected to seed the network connectivity of DMN, TPN and sensory networks (SN). The first ROI set included: the posterior cingulate cortex (PCC) (MNI coordinates: 0, −56, 30) dorsal medial prefrontal cortex (DMPFC) (0, 54, 30) and left angular gyrus (AG) (−45, −66, 30) within the DMN; the second ROI set included: right dorsal lateral prefrontal cortex (DLPFC) (42, 45, 26), left intraparietal lobular (IPL) (−20, −60, 54) and left frontal eye field (FEF) (−26, 2, 52) in the TPN; and the third ROI set included: the left primary somatosensory cortex (SC) (−50, −24, 46), left primary visual cortex (VC) (−20, −84, −4) and left auditory cortex (AC) (−60, −18, 2) in the SN.

#### TS-ICN

At an individual level analysis, a general-linear-model (GLM) in SPM8 was used to calculate the voxel-wise time-series correlation map for each ROI. The head motion parameters, averaged signals from the subject-specific CSF and WM, and the global brain signal were regressed out to remove the possible spurious variances [5], [33]. At a group-level analysis, a random-effect analysis of one-sample *t*-tests (*P*<0.05, FWE corrected) was performed for each ROI-based map. The center, individual gender and age were entered as covariates in group-level analysis.

#### ALFF-FCN

The averaged regional ALFF values within each ROI were extracted from each subject and used as a regressor in the GLM. This analysis produced FCN *t*-map for each ROI, which reflects the covarying ALFF across subjects between a brain region and the seed ROI (i.e., ALFF-FCN). The mean WM and CSF ALFF values, the center, individual gender and age were modeled as covariates in regression analyses (*P*<0.05, Family wise Error [FWE] corrected).

#### VBM-SCN

Similar to the analyses in Zielinski et al. [19] and the procedure of the VBM-SCN computation, the mean values of gray matter volume in each ROI was extracted from each subject, and used as a regressor of the GLM in SPM8 to produce group-level VBM-SCN *t*-maps. The total cranial volume, the whole gray matter volume, the center, the individual gender and age were modeled as covariates in regression analyses (*P*<0.05, FWE corrected).

### Clustering analysis of hierarchical organization of brain networks

To investigate the hierarchical organization of brain networks, a hierarchical clustering analysis was applied to the nine maps of ROI-based networks in ALFF-FCN, TS-ICN and VBM-SCN, respectively. For each pair of ROI-based network maps, the spatial correlation coefficient (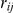) was first transformed into a dissimilarity distance (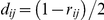), as previously suggested [34]. Then, nine seed-based network maps were hierarchically aggregated according to a minimal dissimilarity cluster distance.

### Conjunction analysis of brain networks

ALFF-FCNs conjunction: Conjunction analysis [5] was used to combine the nine ALFF-FCN maps with similar spatial pattern of network. First, the ALFF-FCN maps were combined within each ROI set. This intra-ROI set conjunction analysis was anticipated to be used for observation of the brain network module patterns. The voxels whose *t* values survived at a threshold at P<0.05 (FWE corrected) were averaged. The average was masked by using a conservative conjunction procedure. Voxels were included in the mask only if they were significantly correlated or anti-correlated with at least 2 of the 3 seed regions. Second, under the guidance by the result of clustering analysis (see section of **Results**), we multiplied the correlation maps of the third ROI set (the sensory system) by −1. Then the correlation maps of the first and the second ROI sets were combined to those of the third ROI set. Voxels were included in the mask only if they were significantly correlated or anti-correlated with at least 8 of the 9 seed regions. The inter-ROI set analysis was anticipated to be used for observation of brain network system patterns.

To compare the patterns network dichotomy between the ALFF-FCN dichotomy and TS-ICN approaches, we repeated the TS-ICN dichotomy in line with the work of Fox et al., [5]. The correlation maps for the DMN ROI sets were multiplied by −1 then averaged with the correlation maps from the TPN ROI sets. Then these two groups of correlation maps were combined to yield the TS-ICN dichotomy consisting of the positive and negative correlations. In addition, the correlation *t*-maps were combined within each ROI set in line with the ALFF-FCNs conjunctions. Since no significant negative correlation and no obvious partition brain network systems (see below) was found in the SCNs, the conjunction analysis was not applied to the SCNs.

## References

[1] Fox MD, Raichle ME (2007). Spontaneous fluctuations in brain activity observed with functional magnetic resonance imaging.. Nat Rev Neurosci.

[2] Zhang D, Raichle ME (2010). Disease and the brain's dark energy.. Nat Rev Neurol.

[3] Raichle ME (2010). The brain's dark energy.. Sci Am.

[4] Greicius MD, Krasnow B, Reiss AL, Menon V (2003). Functional connectivity in the resting brain: a network analysis of the default mode hypothesis.. Proc Natl Acad Sci U S A.

[5] Fox MD, Snyder AZ, Vincent JL, Corbetta M, Van Essen DC (2005). The human brain is intrinsically organized into dynamic, anticorrelated functional networks.. Proc Natl Acad Sci U S A.

[6] Biswal B, Yetkin FZ, Haughton VM, Hyde JS (1995). Functional connectivity in the motor cortex of resting human brain using echo-planar MRI.. Magn Reson Med.

[7] Lowe MJ, Mock BJ, Sorenson JA (1998). Functional connectivity in single and multislice echoplanar imaging using resting-state fluctuations.. Neuroimage.

[8] Fox MD, Greicius M (2010). Clinical applications of resting state functional connectivity.. Front Syst Neurosci.

[9] Mantini D, Perrucci MG, Del Gratta C, Romani GL, Corbetta M (2007). Electrophysiological signatures of resting state networks in the human brain.. Proc Natl Acad Sci U S A.

[10] Greicius MD, Srivastava G, Reiss AL, Menon V (2004). Default-mode network activity distinguishes Alzheimer's disease from healthy aging: evidence from functional MRI.. Proc Natl Acad Sci U S A.

[11] Smith SM, Fox PT, Miller KL, Glahn DC, Fox PM (2009). Correspondence of the brain's functional architecture during activation and rest.. Proc Natl Acad Sci U S A.

[12] He Y, Wang J, Wang L, Chen ZJ, Yan C (2009). Uncovering intrinsic modular organization of spontaneous brain activity in humans.. PLoS One.

[13] Meunier D, Lambiotte R, Bullmore ET (2010). Modular and hierarchically modular organization of brain networks.. Front Neurosci.

[14] Fransson P (2005). Spontaneous low-frequency BOLD signal fluctuations: an fMRI investigation of the resting-state default mode of brain function hypothesis.. Hum Brain Mapp.

[15] Biswal BB, Mennes M, Zuo XN, Gohel S, Kelly C (2010). Toward discovery science of human brain function.. Proc Natl Acad Sci U S A.

[16] Tian L, Jiang T, Liu Y, Yu C, Wang K (2007). The relationship within and between the extrinsic and intrinsic systems indicated by resting state correlational patterns of sensory cortices.. Neuroimage.

[17] Golland Y, Bentin S, Gelbard H, Benjamini Y, Heller R (2007). Extrinsic and intrinsic systems in the posterior cortex of the human brain revealed during natural sensory stimulation.. Cereb Cortex.

[18] Mechelli A, Friston KJ, Frackowiak RS, Price CJ (2005). Structural covariance in the human cortex.. J Neurosci.

[19] Zielinski BA, Gennatas ED, Zhou J, Seeley WW (2010). Network-level structural covariance in the developing brain.. Proc Natl Acad Sci U S A.

[20] Brickman AM, Habeck C, Zarahn E, Flynn J, Stern Y (2007). Structural MRI covariance patterns associated with normal aging and neuropsychological functioning.. Neurobiol Aging.

[21] Bergfield KL, Hanson KD, Chen K, Teipel SJ, Hampel H (2010). Age-related networks of regional covariance in MRI gray matter: reproducible multivariate patterns in healthy aging.. Neuroimage.

[22] He Y, Chen ZJ, Evans AC (2007). Small-world anatomical networks in the human brain revealed by cortical thickness from MRI.. Cereb Cortex.

[23] Zang YF, He Y, Zhu CZ, Cao QJ, Sui MQ (2007). Altered baseline brain activity in children with ADHD revealed by resting-state functional MRI.. Brain Dev.

[24] Zuo XN, Di Martino A, Kelly C, Shehzad ZE, Gee DG (2010). The oscillating brain: complex and reliable.. Neuroimage.

[25] Zou QH, Zhu CZ, Yang Y, Zuo XN, Long XY (2008). An improved approach to detection of amplitude of low-frequency fluctuation (ALFF) for resting-state fMRI: fractional ALFF.. J Neurosci Methods.

[26] Raichle ME, MacLeod AM, Snyder AZ, Powers WJ, Gusnard DA (2001). A default mode of brain function.. Proc Natl Acad Sci U S A.

[27] Yang H, Long XY, Yang Y, Yan H, Zhu CZ (2007). Amplitude of low frequency fluctuation within visual areas revealed by resting-state functional MRI.. Neuroimage.

[28] Zhang Z, Lu G, Zhong Y, Tan Q, Chen H (2010). fMRI study of mesial temporal lobe epilepsy using amplitude of low-frequency fluctuation analysis.. Hum Brain Mapp.

[29] Lui S, Huang X, Chen L, Tang H, Zhang T (2009). High-field MRI reveals an acute impact on brain function in survivors of the magnitude 8.0 earthquake in China.. Proc Natl Acad Sci U S A.

[30] Mennes M, Kelly C, Zuo XN, Di Martino A, Biswal BB (2010). Inter-individual differences in resting-state functional connectivity predict task-induced BOLD activity.. Neuroimage.

[31] Yan C, Zang Y (2010). DPARSF: a MATLAB toolbox for “pipeline” data analysis of resting-state fMRI.. Front Syst Neurosci.

[32] Fair DA, Cohen AL, Dosenbach NU, Church JA, Miezin FM (2008). The maturing architecture of the brain's default network.. Proc Natl Acad Sci U S A.

[33] Fox MD, Zhang D, Snyder AZ, Raichle ME (2009). The global signal and observed anticorrelated resting state brain networks.. J Neurophysiol.

[34] Doucet G, Naveau M, Petit L, Delcroix N, Zago L Brain activity at rest: a multiscale hierarchical functional organization.. J Neurophysiol.

[35] Horwitz B (2003). The elusive concept of brain connectivity.. Neuroimage.

[36] Fingelkurts AA, Fingelkurts AA, Kahkonen S (2005). Functional connectivity in the brain–is it an elusive concept?. Neurosci Biobehav Rev.

[37] Young JP, Geyer S, Grefkes C, Amunts K, Morosan P (2003). Regional cerebral blood flow correlations of somatosensory areas 3a, 3b, 1, and 2 in humans during rest: a PET and cytoarchitectural study.. Hum Brain Mapp.

[38] Horwitz B, Duara R, Rapoport SI (1984). Intercorrelations of glucose metabolic rates between brain regions: application to healthy males in a state of reduced sensory input.. J Cereb Blood Flow Metab.

[39] Friston KJ, Frith CD, Liddle PF, Frackowiak RS (1993). Functional connectivity: the principal-component analysis of large (PET) data sets.. J Cereb Blood Flow Metab.

[40] Lee DS, Kang H, Kim H, Park H, Oh JS (2008). Metabolic connectivity by interregional correlation analysis using statistical parametric mapping (SPM) and FDG brain PET; methodological development and patterns of metabolic connectivity in adults.. Eur J Nucl Med Mol Imaging.

[41] Blumenfeld H, McNally KA, Vanderhill SD, Paige AL, Chung R (2004). Positive and negative network correlations in temporal lobe epilepsy.. Cereb Cortex.

[42] Seeley WW, Crawford RK, Zhou J, Miller BL, Greicius MD (2009). Neurodegenerative diseases target large-scale human brain networks.. Neuron.

[43] Hasson U, Nir Y, Levy I, Fuhrmann G, Malach R (2004). Intersubject synchronization of cortical activity during natural vision.. Science.

[44] Yan C, Liu D, He Y, Zou Q, Zhu C (2009). Spontaneous brain activity in the default mode network is sensitive to different resting-state conditions with limited cognitive load.. PLoS One.

[45] Gaab N, Gabrieli JD, Glover GH (2008). Resting in peace or noise: scanner background noise suppresses default-mode network.. Hum Brain Mapp.

[46] Mantini D, Caulo M, Ferretti A, Romani GL, Tartaro A (2009). Noxious somatosensory stimulation affects the default mode of brain function: evidence from functional MR imaging.. Radiology.

[47] Greicius MD, Flores BH, Menon V, Glover GH, Solvason HB (2007). Resting-state functional connectivity in major depression: abnormally increased contributions from subgenual cingulate cortex and thalamus.. Biol Psychiatry.

[48] Delamillieure P, Doucet G, Mazoyer B, Turbelin MR, Delcroix N (2010). The resting state questionnaire: An introspective questionnaire for evaluation of inner experience during the conscious resting state.. Brain Res Bull.

[49] Murphy K, Birn RM, Handwerker DA, Jones TB, Bandettini PA (2009). The impact of global signal regression on resting state correlations: are anti-correlated networks introduced?. Neuroimage.

[50] Van Dijk KR, Hedden T, Venkataraman A, Evans KC, Lazar SW (2010). Intrinsic functional connectivity as a tool for human connectomics: theory, properties, and optimization.. J Neurophysiol.

[51] Weissenbacher A, Kasess C, Gerstl F, Lanzenberger R, Moser E (2009). Correlations and anticorrelations in resting-state functional connectivity MRI: A quantitative comparison of preprocessing strategies.. Neuroimage.

